# Soil conditioning effects of *Phragmites australis* on native wetland plant seedling survival

**DOI:** 10.1002/ece3.3024

**Published:** 2017-06-15

**Authors:** Ellen V. Crocker, Eric B. Nelson, Bernd Blossey

**Affiliations:** ^1^ Department of Plant Pathology Cornell University Ithaca NY USA; ^2^ Department of Natural Resources Cornell University Ithaca NY USA

**Keywords:** invasion ecology, plant traits, plant–soil (belowground) interactions, plant–soil feedbacks, seedling survival, soil conditioning, wetlands

## Abstract

Interactions between introduced plants and soils they colonize are central to invasive species success in many systems. Belowground biotic and abiotic changes can influence the success of introduced species as well as their native competitors. All plants alter soil properties after colonization but, in the case of many invasive plant species, it is unclear whether the strength and direction of these soil conditioning effects are due to plant traits, plant origin, or local population characteristics and site conditions in the invaded range. *Phragmites australis* in North America exists as a mix of populations of different evolutionary origin. Populations of endemic native *Phragmites australis americanus* are declining, while introduced European populations are important wetland invaders. We assessed soil conditioning effects of native and non‐native *P. australis* populations on early and late seedling survival of native and introduced wetland plants. We further used a soil biocide treatment to assess the role of soil fungi on seedling survival. Survival of seedlings in soils colonized by *P. australis* was either unaffected or negatively affected; no species showed improved survival in *P. australis*‐conditioned soils. Population of *P. australis* was a significant factor explaining the response of seedlings, but origin (native or non‐native) was not a significant factor. *Synthesis*: Our results highlight the importance of phylogenetic control when assessing impacts of invasive species to avoid conflating general plant traits with mechanisms of invasive success. Both native (noninvasive) and non‐native (invasive) *P. australis* populations reduced seedling survival of competing plant species. Because soil legacy effects of native and non‐native *P. australis* are similar, this study suggests that the close phylogenetic relationship between the two populations, and not the invasive status of introduced *P. australis*, is more relevant to their soil‐mediated impact on other plant species.

## 1 INTRODUCTION

Interactions between introduced plant species and the soils they colonize are increasingly being recognized for their central role in determining success and failure of plants to establish, grow, and become invasive (Hierro & Callaway, 2003; Inderjit & van der Putten, 2010; Mitchell et al., 2006; Reinhart & Callaway, 2006; Wolfe & Klironomos, 2005). Although all plants have species‐specific effects on soil they colonize (Bardgett & van der Putten, 2014; Berg & Smalla, 2009), invasive plants often appear to alter soils to their advantage, creating positive plant–soil feedback and promoting dominance (Berg & Smalla, 2009; Bever, 1994; Diez et al., 2010; Fitzsimons & Miller, 2010; Flory & Clay, 2013; Klironomos, 2002; Kowalchuk, Buma, De Boer, Klinkhamer, & van Veen, 2002; Peterman, Fergus, Turnbull, & Schmid, 2008; van der Putten et al., 2013; van Grunsven et al., 2007). Soil biota contribute strongly to these plant–soil feedbacks, and seedling survival appears to be a critical demographic stage in determining invasive success (Blaney & Kotanen, 2001; Packer & Clay, 2000; Reinhart & Clay, 2009; Reinhart, Tytgat, van der Putten, & Clay, 2010). Sometimes invasive plant seedlings are less susceptible to soil pathogens (Reinhart et al., 2010b). Alternatively, they may be able to condition soil resulting in increased disease incidence on competing seedling (Beckstead, Meyer, Connolly, Huck, & Street, 2010).

Understanding soil legacy effects is of considerable importance for conservation and management of plant invasions. Removal of introduced species may alleviate their impact on resource competition above and below ground, but if their soil conditioning legacies continue, they may impede successful site restoration (Suding, Gross, & Houseman, 2004; Suding & Hobbs, 2009; Yelenik & Levine, 2010). Furthermore, recent studies establish the importance of genotypes and plant functional traits as strong influences on soil biota (van der Putten et al., 2013), and their effect on associated consumers, such as amphibians (Martin & Blossey, 2013a). Intraspecific variation in plant species and genotypes in their effects on soils may affect soil biota community composition, which, in turn, can affect aboveground plant community composition (van der Putten et al., 2013). The ecological and evolutionary dynamics and impacts of these interactions have only recently become the focus of investigations in nonagricultural systems. Further evaluation of impacts and mechanisms associated with the purposeful and accidental movement and spread of potentially invasive species would greatly enhance our ability to understand and potentially manage recovery and conservation of rare or declining species that appear to suffer the most from negative soil feedbacks.

We were interested in assessing how wetland plant communities in North America, particularly at the seedling stage, are affected by introduced genotypes of *Phragmites australis* and the role soil microbial communities may have in determining the outcome of these interactions. Introduced from Europe, *P. australis* is one of the most important invasive plants spreading through North America, forming dense monocultures along roadsides, in tidal areas and in wetlands (Chambers, Meyerson, & Saltonstall, 1999; Saltonstall, 2002). The spread of introduced *P*. *australis*, hereafter referred to as EU, is unique as endemic native haplotypes, recently elevated to subspecies level *Phragmites australis americanus* (Saltonstall, Peterson, & Soreng, 2004) and hereafter referred to as NA*,* are widespread on the continent but are being replaced by advancing European genotypes (Meadows & Saltonstall, 2007; Saltonstall, 2002, 2003). There are large overall similarities in growth pattern and other traits between native and introduced genotypes (Martin & Blossey, 2013a; Park & Blossey, 2008), but native genotypes are generally considered noninvasive, although certain populations can rapidly expand (Lynch & Saltonstall, 2002). Observational evidence suggests that floristic diversity is higher in NA stands in contrast to dense near monospecific stands of EU, which are of great concern to wetland managers. These concerns have led to widespread and extensive herbicide control campaigns targeting EU populations that are largely unsuccessful (Martin & Blossey, 2013b), prompting an attempt to develop a biological control program (Tewksbury, Casagrande, Blossey, Häfliger, & Schwarzländer, 2002).

The advancement of EU involves long‐distance dispersal via short‐lived seed and rhizome fragments as well as local clonal spread through rhizomes (Belzile, Labbé, Leblanc, & Lavoie, 2010; Chambers, Osgood, Bart, & Montalto, 2003; Chambers et al., 1999; Jodoin et al., 2008; McCormick, Kettenring, Baron, & Whigham, 2010a,b). Seedling establishment is key to the success of EU (Belzile et al., 2010)*,* NA, and other native plant species, which typically regenerate from long‐lived seed banks often on exposed mudflats after water draw downs (van der Valk, 1981; van Grunsven et al., 2007), although EU seeds are very short‐lived. Suggestions that EU may produce root‐secreted allelopathic gallic acid inhibiting other plant species (Bains et al., 2009; Galatowitsch, Anderson, & Ascher, 1999; Rudrappa, Bonsall, Gallagher, Seliskar, & Bais, 2007; Zedler & Kercher, 2004) are contested (Weidenhamer, Li, Allman, Bergosh, & Posner, 2013), yet overall soil legacy effects of introduced EU on germination and seedling recruitment of native competitors remain unclear.

We established a common garden and a field transplant experiments to assess soil conditioning effects that may contribute to the success of NA and EU on early (first 4 weeks including germination) and late (2 months after germination) seedling survival of native plant species. We tested the following hypotheses: (1) Successful germination and early seedling survival will be higher in soils conditioned by NA than in soils conditioned by EU*—*an origin effect*;* (2) fungicide application will eliminate negative soil conditioning effects of EU; and (3) in the field, soils conditioned by EU will reduce seedling survival compared to seedling survival in the surrounding wetland species matrix.

## 2 METHODS

### 2.1 Germination and early seedling survival

In summer of 2008, we established a common garden at the Cornell Resource Ecology and Management Facility (REM) in Ithaca, New York, growing EU and NA *P. australis* populations from field‐collected rhizome fragments in 10‐m‐long, 50‐cm‐wide, and 50‐cm‐deep trenches lined with pond liner (45 mil EPDM [ethylene propylene diene monomer], Pondliner.com, Shawnee, Oklahoma) and filled with Cornell compost mix (Cornell University, Ithaca, NY, USA). We propagated plants from rhizome cuttings obtained from Maine (ME), Minnesota (MN), Indiana (IN), New York (NY), South Dakota (SD), and Washington (WA) (see Table S1 and Fig. S1), hereafter referred to as populations. Within each area, we were able to pair collection locations because NA and EU existed within a short distance from each other, allowing us to reduce effects of longitudinal and latitudinal influences on our results. We used reliable morphological features to assign populations to EU or NA, and all populations were further assigned to haplotypes (see Saltonstall, 2002, but haplotype information was not used in this experiment). To reduce potential environmental effects of field collection location via maternal effects, we propagated field‐collected rhizome cuttings for 2 years in a common garden in 100‐L tree pots (BFG Supply, Lancaster, NY, USA) filled with commercial potting soil (Fafard Canadian growing mix No. 1‐P, Agawam, MA, USA). In 2008, we obtained fresh rhizome cuttings from these pot‐grown plants for our trench experiment. We completely randomized planting locations within our common garden and established five replicate trenches for each population and allowed plants to expand through clonal growth within their trenches (other plants were regularly removed) until they were well established.

Two years after planting into the trenches, we sampled soil from the rhizosphere of three to five trenches per population on August 11, 2010, and homogenized samples for each population. We also collected control soils in trenches that remained without any NA or EU *P. australis* growth but were otherwise treated in an identical manner. After homogenizing, we filled 107‐ml individually labeled plastic containers (Ray Leach Cone‐tainer SC7U, Tangent, Oregon) with soil from each population, and arranged them randomly in plastic trays (98 containers/tray) that kept containers 5 cm off the ground and 2 cm apart. On August 12, 2010, we treated half of the containers from each population and half of the unconditioned control containers with a broad‐spectrum nonsystemic fungicide, Daconil Weather Stik^®^ (active ingredient chlorothalonil, Syngenta, Greensboro, North Carolina), at the highest recommended single field application rate (0.125 mg active ingredient/cm^2^). We randomly arranged trays outdoors in a walk‐in field cage (Lumite^®^ screening, shade 15%, porosity 1629CFM; Synthetic Industries, Gainesville, GA, USA), exposing seeds to outside fluctuating summer conditions but preventing bird or mammal disturbances, and rearranged containers every week.

We purchased seed of *Asclepias incarnata* (swamp milkweed), *Astragalus canadensis* (Canadian milkvetch)*, Calamagrostis canadensis* (bluejoint grass), *Carex lacustris* (lake sedge), *Epilobium glandulosum* (northern willowherb), *Eupatorium maculatum* (spotted Joe‐Pye weed)*, Euthamia graminifolia* (grass‐leaved goldenrod), and *Juncus effusus* (common rush) from Prairie Moon, Winona, Minnesota, and *Phalaris arundinacea* (reed canarygrass) from River Source Botanical, Taos, New Mexico. Where necessary, we cold‐stratified seeds according to grower's directions. All species show high seed viability and very rapid germination under suitable conditions (Baskin, 1998). On August 15, 2010, we planted five or 20 seeds per species (Table S2) into each container. The difference in number of seeds was based on expected early seedling size. As designed, this experiment integrates the combined effects of potential suppression of germination, very early death upon germination but before emergence of cotyledon, as well as very early seedling mortality before seedlings can be called established. Except for the death of seedlings with established aboveground shoots, this mortality is often difficult to observe. We established 10 replicate containers for each combination of species/soil type/fungicide treatment for a total of 1980 containers (11 soil types [5 NA, 5 EU, 1 control *P. australis*‐free soil] × 2 soil treatments [none, fungicide] × 9 plant species × 10 replicate containers). We watered containers every three to 5 days, recorded the number of surviving seedlings every 5 days, and scored final seedling survival on September 15, 2010, when we terminated the experiment.

### 2.2 Transplant survival

We assessed EU effects on wetland seedling survival and growth at four field sites in the Montezuma Wetlands Complex, Savannah NY (see Table S3). We were unable to include NA impacts in this experiment because existing stands in the study area are small and growing intermixed with other plant species preventing us from isolating NA‐specific effects. We selected locations with dense EU populations adjacent to mixed wetland plant communities. At each site, we located four 3 × 3 m plots, two in the interior (at least 5 m inside from the edge of the EU stand), and two in diverse marsh vegetation at least 5 m away from the edge of the EU invasion front. Plots at each site were within 50 m of each other, and all sites were within 10 km of each other. At each plot, we removed all aboveground vegetation using clippers and cleared the area of leaf litter to expose the wetland soil surface. This treatment kept belowground rhizomes and roots intact but eliminated potentially confounding effects of light competition on seedling survival. We continued to weed experimental areas weekly by hand using clippers to minimize soil disturbance until the termination of the experiment.

We propagated seedlings of seven plant species of which five (*A. incarnata, A. canadensis*,* C. canadensis*,* E. glandulosum*, and *E. graminifolia*) were also used for the early seedling growth experiment (Table S2). We purchased seed of *Elymus riparius* (riverbank wildrye), *Mimulus ringens* (monkeyflower), and *Muhlenbergia glomerata* (marsh muhly) from Prairie Moon, Winona, Minnesota, USA. These plants span a wide phylogenetic range and are easy to propagate. We followed species‐specific germination requirements and grew plants until they were approximately 2 months old in a glasshouse at Cornell University in a potting mix/sand media.

**Figure 1:**
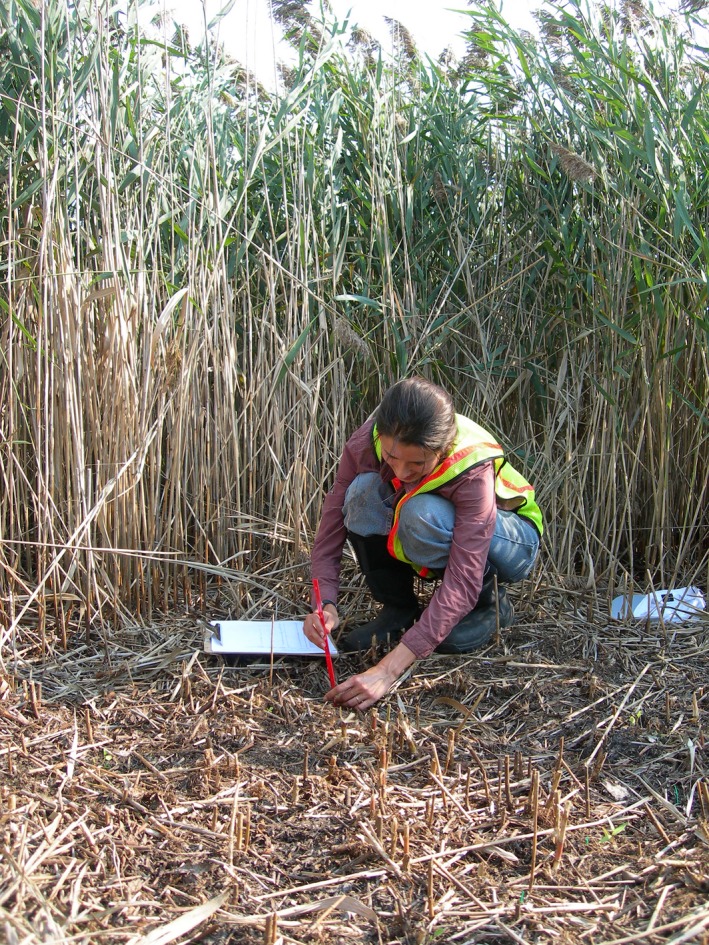
Organismal photograph of *Phragmites australis* (EU). Photograph shows assessment of seedlings transplanted into EU patches. Photograph credit Allison Jack

Before field transplanting, we established a grid (1.8 m × 1.8 m, cell size 20 cm × 20 cm) in June 2011 in each cleared plot and randomly planted 1120 individuals (10 individuals × 7 plant species × 4 plots per site × 4 sites). For each plot, we selected similar sized individuals of each plant species and randomly assigned them to specific cells. We planted sites on consecutive days to minimize drying while waiting to be transplanted. We watered plants weekly due to an extended drought at all field sites in summer 2011 and assessed plant survival at 4 weeks after transplanting (Figure 1).

### 2.3 Analyses

To test the influence of soil properties on both early seedling common garden and late seedling field survival, we employed generalized linear mixed models (GLMMs) with binomial distribution. For early seedling survival in common garden soil, we tested main effects of *P. australis* conditioning (NA, EU, or *P. australis*‐free control), origin (NA or EU), and soil fungicide (treated or not) on seedling survival at 4 weeks. We included collection location as a random variable and ran separate analyses for each plant species. We evaluated differences between EU and NA a posteriori by aggregating both initially (Crawley, 2012). Starting with the full model (which included *P. australis* soil conditioning [control*, P. australis*], soil fungicide application, and their interaction), we reduced models in a backwards stepwise process to determine the best model and significance via log‐likelihood tests at *p *< .05. For late seedling survival, we tested main effects of EU colonization (invaded and noninvaded), with site as a random variable, and followed the same backwards stepwise process to determine the best model. We used R version 3.0.1 (R Development Core Team, 2013) and the add‐on package “lme4” (Bates, Maechler, Bolker, & Walker, 2013).

## 3 RESULTS

### 3.1 Germination and early seedling survival

**Figure 2:**
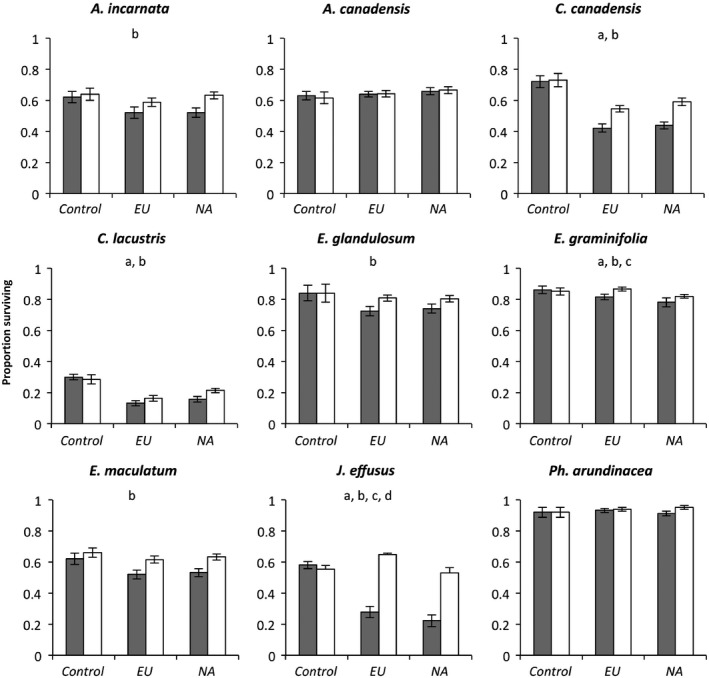
Proportion early seedling survival of nine plant species when sown onto experimental soils (soil types: *Phragmites australis*‐free control, conditioned by introduced *P*. *australis* [EU], conditioned by native *Phragmites australis americanus* [NA], untreated soil (gray bars), and fungicide‐treated soil (white bars). Data are means ± 1*SE* with either 10 (control soils) or 50 (all other treatments) replicates

Germination and early seedling survival were extremely variable among the different plants species ranging from >80% for *P. arundinacea* to <20% for *C. lacustris* (Figure 2), and there was a strong effect of population (Fig. S2). Soil conditioning by NA and EU reduced survival for four of nine species (*C. canadensis*,* C. lacustris*,* E. graminifolia*, and *J. effusus*), but the others (*A. incarnata*,* A. canadensis, E. glandulosum*,* E. maculatum*, and *P. arundinacea*) remained unaffected (Figure 2, Table 1). Effects of EU and NA were either negative or neutral but never increased survival of the tested wetland species (Figure 2, Table 1). For two species, *E. graminifolia* and *J. effusus*, we found a significant origin effect, but reductions in survival were larger when soils were conditioned by NA compared to EU or *P. australis*‐free control soils (Figure 2, Table 1). Fungicide application had no impact on seedling survival in control soils but increased survival in conditioned soils for all species except for *A. canadensis* and *P. arundinacea* (Figure 2, Table 1). We found a significant interaction of fungicide application and origin (NA or EU) for *J. effusus* with fungicide increasing survival in EU‐conditioned soils more than in NA‐conditioned soils (Figure 2, Table 1).

**Table 1:** Model results for effect of *Phragmites australis* presence on seedling survival for nine plant species in *P. australis‐*conditioned soil analyzed using a generalized linear mixed model (GLMM) with binomial distribution. Models included fungicide treatment and soil conditioning (*P. australis*‐free control, introduced EU/*P. australis*, or native NA/*Phragmites australis americanus* a) as fixed effects and collection location as a random effect^b^

Species	Intercept	Fungicide (F)	Soil conditioning (S) EU and/or NA	F × S interaction
*Asclepias incarnata*	0.13 ± 0.15	0.35 ± 0.12**		
*Astragalus canadensis*	0.61 ± 0.05			
*Calamagrostis canadensis*	0.73 ± 0.29	0.53 ± 0.09***	−0.99 ± 0.31**	
*Carex lacustris*	−1.03 ± 0.61	0.28 ± 0.08***	−0.87 ± 0.66**	
*Epilobium glandulosum*	1.12 ± 0.19	0.40 ± 0.15**		
*Eupatorium maculatum*	0.14 ± 0.09	0.38 ± 0.12**		
*Euthamia graminifolia*	1.66 ± 0.11	0.27 ± 0.08***	−0.1 ± 0.24*** (NA) −0.39 ± 0.24*** (EU)	
*Juncus effusus*	0.32 ± 0.59	−0.10 ± 0.20	−1.36 ± 0.65 (EU) −1.68 ± 0.65** (NA)	1.80 ± 0.23*** (EU) 1.60 ± 0.23*** (NA)
*Phalaris arundinacea*	2.63 ± 0.12			

^a^ EU and NA combined unless origin is significant.

^b^Empty cells denote parameters that were not part of the best model.

Asterisks indicate *p*‐values from log‐likelihood tests between a model without the term and a model with all terms included (*p < .05; **p < .01, ***p < .001).

### 3.2 Transplant survival

**Figure 3:**
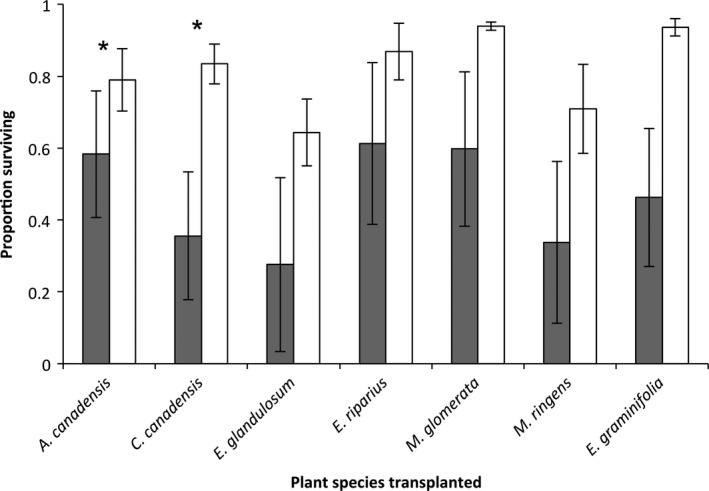
Probability of survival when transplanted into introduced *Phragmites australis* (EU, gray bars) or the adjacent wetland plant community (white bars) for seven different plant species. Data are means of each species tested (*n* = 4 sites; 10 individuals/species in each of 4 plots/site). Asterisk (*) indicates significant differences (GLMM,* p* < .05)

Transplant survival varied dramatically among species and locations (Figure 3) with all species showing 60%–80% survival when transplanted into a mixed‐species wetland matrix. In contrast, transplant survival was reduced for all species when growing inside EU, but differences in survival were only significant for *A. canadensis* and *C. canadensis* (Figure 3, Table 2). Transplant survival within EU stands differed widely by site, with the lowest survival at Carncross (9%, mean of all species) but very high at Teal Pond (94%, mean of all species) (Fig. S3).

**Table 2:** Model results for effect of introduced *Phragmites australis*/EU presence on seedling survival for seven plant species analyzed using a generalized linear mixed model (GLMM) with binomial distribution. Models included EU presence (P) as fixed effects and site as a random effect

Species	Intercept	EU presence
*Astragalus canadensis*	0.33 ± 0.49	1.58 ± 0.52*
*Calamagrostis canadensis*	−0.91 ± 0.75	2.85 ± 0.74*
*Epilobium glandulosum*	−0.35 ± 1.16	
*Elymus riparius*	1.56 ± 0.87	
*Euthamia graminifolia*	1.46 ± 0.86	
*Muhlenbergia glomerata*	1.83 ± 0.95	
*Mimulus ringens*	0.16 ± 0.94	

We determined significance of each factor in a best model by log‐likelihood comparisons of best model and model missing each factor (*p < .05; **p < .01, ***p < .001).

## 4 DISCUSSION

We designed our common garden and field experiments to assess potential mechanisms contributing to invasiveness (measured as suppression of other wetland seedlings) of EU in North America using plant–soil feedback theory (PSF). PSF theory predicts that invasive plant species can engineer a competitive advantage over native plants through soil conditioning effects (Beckstead et al., 2010; Eppinga, Rietkerk, Dekker, & De Ruiter, 2006; Hierro & Callaway, 2003; Mangla & Callaway, 2007; Mitchell et al., 2006; Reinhart & Callaway, 2006; Saltonstall, 2003; Wolfe & Klironomos, 2005). Our results demonstrate that both EU and NA may amplify the abundance, or facilitate the colonization, of fungi that reduce survival of competing wetland plant species at the seedling stage. This is clearly a soil conditioning effect in response to active plant growth as control soils (those without *P. australis* growth) do not show this effect (Figure 2). However, this effect is not restricted to EU as would be expected with PSF theory as its native congener, NA, has similar negative impacts on survival of other wetland plant seedlings (Figures 2 and S2). In contrast to our hypothesis, the strength of suppression does not depend on origin. Furthermore, even for those species for which origin was a significant factor (*E. graminifolia* and *J. effusus*), effect sizes in the common garden study were small, questioning their ecological relevance in affecting plant community dynamics. Although we were unable to incorporate origin effects into our transplant study, lack of significance in survival between individuals growing inside and outside of EU patches suggests that PSF theory and soil conditioning effects alone appear to have little power to explain invasiveness of EU and lack of plant diversity in established stands.

Although our experiments covered only the very early life history of wetland species, and the potential for effects to change or magnify over time does exist, our results suggest that invasive success of EU does not depend on unique soil conditioning mechanisms that distinguish it from NA. These results support other studies reporting trait similarities between NA and EU (Park & Blossey, 2008; Saltonstall, 2002) as well as effects on consumers (Larochelle, Dumont, Lavoie, & Hatin, 2014; Martin & Blossey, 2013a). However, field evidence clearly shows rapidly expanding populations and development of near monocultures of EU across many coastal and inland wetlands in North America (Saltonstall, 2002, 2003). In our attempt to assess PSF mechanisms, we eliminated effects of resources competition, for example, for light and nutrients by cutting aboveground vegetation. Yet plant height and clonal extent are important factors in determining competitive hierarchies in wetland plant communities (Gaudet & Keddy, 1988; Keddy & Shipley, 1989; Keddy, Twolan‐Strutt, & Wisheu, 1994). Various other factors, including superior photosynthetic capacity (Mozdzer & Zieman, 2010), suppression of competitors by shade and litter (Haslam, 1971a,b; Holdredge & Bertness, 2010; Minchinton, Simpson, & Bertness, 2006), shoreline development and eutrophication (Bertness, Ewanchuk, & Silliman, 2002; Holdredge, Bertness, & von Wettberg, 2010), as well as effect of consumers such as herbivorous crabs (Holdredge, Bertness, & Altieri, 2008), are frequently mentioned to explain the invasive success of EU. In contrast, some experimental evidence suggests that functional group identity and diversity of resident plant communities may represent a form of biotic resistance (Byun, de Blois, & Brisson, 2012).

Our results establish the importance of soil fungal communities, EU or NA population (but not origin), and growing location on survival of different wetland competitor species, at least at an early life stage. This was especially prominent in our transplant experiment where seedling survival at one site was unaffected by EU invasion. Although this may be a function of site‐specific conditions, EU colonization at this site may also be more recent and not have accumulated negative soil feedbacks (Diez et al., 2010; Meadows & Saltonstall, 2007; Packer & Clay, 2004). Understanding how soil fungal communities interact with other factors reported to facilitate EU invasion will require more detailed, and more long‐term, studies that go beyond the typical experimental investigations of a few years but may offer some intriguing potential for invasion management.

Our results of fungicide treatments suggest that the importance of soil fungi in seedling establishment is consistent with many other studies that point to soil fungal pathogens as key contributors to soil legacy effects (Klironomos, 2002; Lynch & Saltonstall, 2002). However, identities of soil fungi that may contribute to reduced seedling survival remain unknown. Although EU is known to host diverse assemblages of fungi (Angelini et al., 2012; Fischer & Rodriguez, 2013; Neubert, Mendgen, Brinkmann, & Wirsel, 2006; Wirsel, Leibinger, Ernst, & Mendgen, 2001), their specific roles in limiting seedling survival are largely unknown. Similarly, EU and NA also associate with diverse communities of oomycetes (Belzile et al., 2010; Chambers et al., 1999, 2003; Jodoin et al., 2008; McCormick et al., 2010a,b; Nechwatal & Mendgen, 2006; Nechwatal, Wielgoss, & Mendgen, 2005, 2008; Nelson & Karp, 2013) that are known to affect plant survival and would have been inhibited by the broad‐spectrum fungicide used. It is important to note that soil bacteria were not investigated here although they may play an important role in soil conditioning. Fungicide treatment is unlikely to have directly altered bacteria in the soil but could have indirectly shifted bacterial community structure or abundance (Smith, Hartnett, & Rice, 2000); however, we did not assess these changes.

The responses of plants to *P. australis* soil conditioning, especially to decreases in soil fungi from fungicide application, varied dramatically among species. Among other wetland competitors, the only other invasive species, *P*.* arundinacea*, was not affected by the fungicide treatment. Others have suggested that invasive success may be conveyed by resistance to soil pathogens (Belzile et al., 2010; Reinhart, Royo, Kageyama, & Clay, 2010). However, *A. canadensis*, a native species, also was not affected by the fungicide treatment, suggesting that soil legacies are species specific and do not follow plant origin.

Our experiments show that soil conditioning by *P. australis* has a significant negative impact on the seedling survival of many plant species, particularly in the very early stages. Because of their impact on seedling survival, soil legacy effects, caused by changes to soil biota or to other abiotic soil properties, likely contribute to plant population dynamics. However, given that these effects are not necessarily determined by whether lineages are considered invasive or not, but appear a function of population or genotype, it is unclear how much PSF contributes to suppression of native plant species and advancement of EU. Does EU cultivate a fungal community that selects for establishment of particular plant species over others? Does shoot height and the clonal nature of the species interact with soil legacy effects in determining plant communities, including after control attempts that typically use herbicides but always fail to suppress the species long term (Martin & Blossey, 2013b)? However, we cannot exclude the possibility that EU creates a “halo” effect where soil conditioning through individual belowground rhizomes may reach beyond the visibly established clonal front. Such a plant community structuring effect was found, although not assessed through a PSF framework for Japanese knotweed, *Fallopia* spp. (Maerz, Blossey, & Nuzzo, 2005), reaching out to near 10 m beyond the invasion front. We established our plots 5 m from the visible aboveground invasion front and this may contributed to the local nonsignificance of transplant survival, but cannot explain the dramatic survival differences between sites (Fig. S3).

Further understanding how intraspecific and lineage traits interact with local soil conditions, human‐facilitated local legacies, and soil community composition seems an important endeavor if we continue to engage in invasive species management (Buckley & Catford, 2016). Origin, considered for a long time an easy trait indicating potential for invasiveness and undesirable impact, fails and not only in our current work (Martin & Blossey, 2013b). Land management practices should consider incorporating roles of soil biota and soil legacy effects as they engage in vegetation management and not rely on origin alone (Perkins & Hatfield, 2016). It will not be an easy task but without evidence‐guided work, the desired outcomes will remain elusive.

## CONFLICT OF INTEREST

None declared.
