# Supplementary material for: Soil conditioning effects of Phragmites australis on native wetland plant seedling survival
Source: Ecol Evol. 2017 Jun 15;7(15):5571–9. doi: 10.1002/ece3.3024 (PMC5551089; doi:10.1002/ece3.3024)
Supplement: Supplementary file 1 [file ECE3-7-5571-s001.docx]

**SUPPORTING INFORMATION**

**Table S1** Collection locations of the *P. australis* (EU and NA) populations.

**Table S2** Plant species and number of seeds used in early seedling common garden (1) and late seedling field transplant (2) experiments.

**Table S3** Site location and vegetation community composition for field transplant survival experiment.

**Figure S1** Map of collection locations of the *P. australis* populations.

**Figure S2** Early seedling survival (%) of different plant species in control soils and soils conditioned by different populations of EU and NA*.*

**Figure S3** Proportion late-seedling survival when transplanted a) at each site outside and within EU patches

**Table S1:** Collection location, state, origin (EU = introduced *P. australis* or NA = native *P. australis americanus*) and Lat/Long of *P. australis* populations established in a common garden. Two-letter code indicates US state (ME = Maine; MN = Minnesota; IN=Indiana, NY = New York; SD = South Dakota; WA = Washington).

| **Collection location** | **State** | **Origin** | **Latitude** | **Longitude** |
| --- | --- | --- | --- | --- |
| Libby River | ME | EU | 43.55 | -70.32 |
| Libby River | ME | NA | 43.55 | -70.32 |
| Forest Lake | MN | EU | 45.28 | -92.99 |
| Pipewort Pond | IN | NA | 41.74 | -85.82 |
| Rochester | NY | EU | 43.11 | -77.73 |
| Bergen Swamp | NY | NA | 43.09 | -77.98 |
| Davison County | SD | EU | 43.60 | -98.11 |
| Clark County | SD | NA | 44.81 | -97.72 |
| Moses Lake | WA | EU | 47.12 | -119.29 |
| Sun Lake | WA | NA | 47.64 | -119.24 |

**Table S2:** Plant species and number of seeds used in early-seedling common garden (1) and species used in field transplant experiments (2).

| **Species** | **Experiment** | **Seeds/ Container** |
| --- | --- | --- |
| *Asclepias incarnata* | 1, 2 | 5 |
| *Astragalus canadensis* | 1, 2 | 20 |
| *Calamagrostis canadensis* | 1, 2 | 10 |
| *Carex lacustris* | 1 | 20 |
| *Elymus riparius* | 2 | - |
| *Epilobium glandulosum* | 1, 2 | 5 |
| *Eupatorium maculatum* | 1 | 5 |
| *Euthamia graminifolia* | 1, 2 | 20 |
| *Juncus effusus* | 1 | 20 |
| *Mimulus ringens* | 2 | - |
| *Muhlenbergia glomerata* | 2 | - |
| *Phalaris arundinacea* | 1 | 5 |

**Table S3**: Site location and vegetation community composition for field transplant survival experiment where species were transplanted into and adjacent to an introduced *P. australis* (EU) population.

| **Location** | **Dominant vegetation outside EU stand** | **Latitude and longitude** |
| --- | --- | --- |
| Martin’s Marsh 1 | Plot 1:  *Equisetum sp* 40%, *Phalaris arundinacea* 20%, *Apocynum cannabinum* 15%, *Lythrum salicaria*, *Carex cristatella*, *Typha*, unknown herbaceous, unknown grass all <5%  Plot 2:  *Equisetum* sp 30%, *Phalaris arundinacea* 30%  *Spartina pectinata* 20%, *Lythrum salicaria* 10%,  *Carex cristatella*, *Carex vulpinoidea*, *Eupatorium maculatum,* *Typha*, unknown herbaceous, unknown grass all <5% | 43° 5'1.69" N  76°42'36.66"W |
| Martin’s Marsh 2 | Plot 1:  *Carex lacustris* 30%, *Solidago sp*. 15%,  *Lysimachia terrestris*, *Spartina pectinata*, *Juncus inflexus*, *Carex cryptolepis*, *Asclepias incarnata*, *Lythrum salicaria, Alnus serrulata, Eupatorium maculatum, Equisetum, Vitis riparia, Phalaris arundinacea*, unknown grasses, unknown herbaceous all <5% | 43° 5'2.86"N  76°42'23.78"W |
| Carncross | Plot 1:  *Eupatorium maculatum* 70%, *Phalaris arundinacea* 20%, *Carex lacustris* <5%, *Solanum dulcamara* <5%  Plot 2:  *Carex lacustris* 50%, *Phalaris arundinacea* 20%, *Eupatorium maculatum* 10%, *Vitis riparia, Apocynum cannabinum, Galium, Solanum dulcamara, Parthenocissus quinquefolia*, unknown herbaceous all <5% | 43°04´56.624´´ N 76°42´38.241´´ W |
| Teal Pond | Plot 1:  *Persicaria maculosa* 50%, *Lythrum salicaria* 40%,  *Carex comosa, Apocynum cannabinum, Phalaris arundinacea*, unknown grasses all <5%  Plot 2:  *Persicaria maculosa* 40%, *Lythrum salicaria* 40%,  *Carex cristatella, Apocynum cannabinum, Phalaris arundinacea, Spartina pectinata, Scirpus cyperinus, Typha sp*. all <5% | 43° 5'9.95"N  76°42'18.26"W |

**Figure S1.** Map of collection locations of the *P. australis* populations. Two-letter code indicates US state (ME = Maine; MN = Minnesota; IN = Indiana, NY = New York; SD = South Dakota; WA = Washington).

**
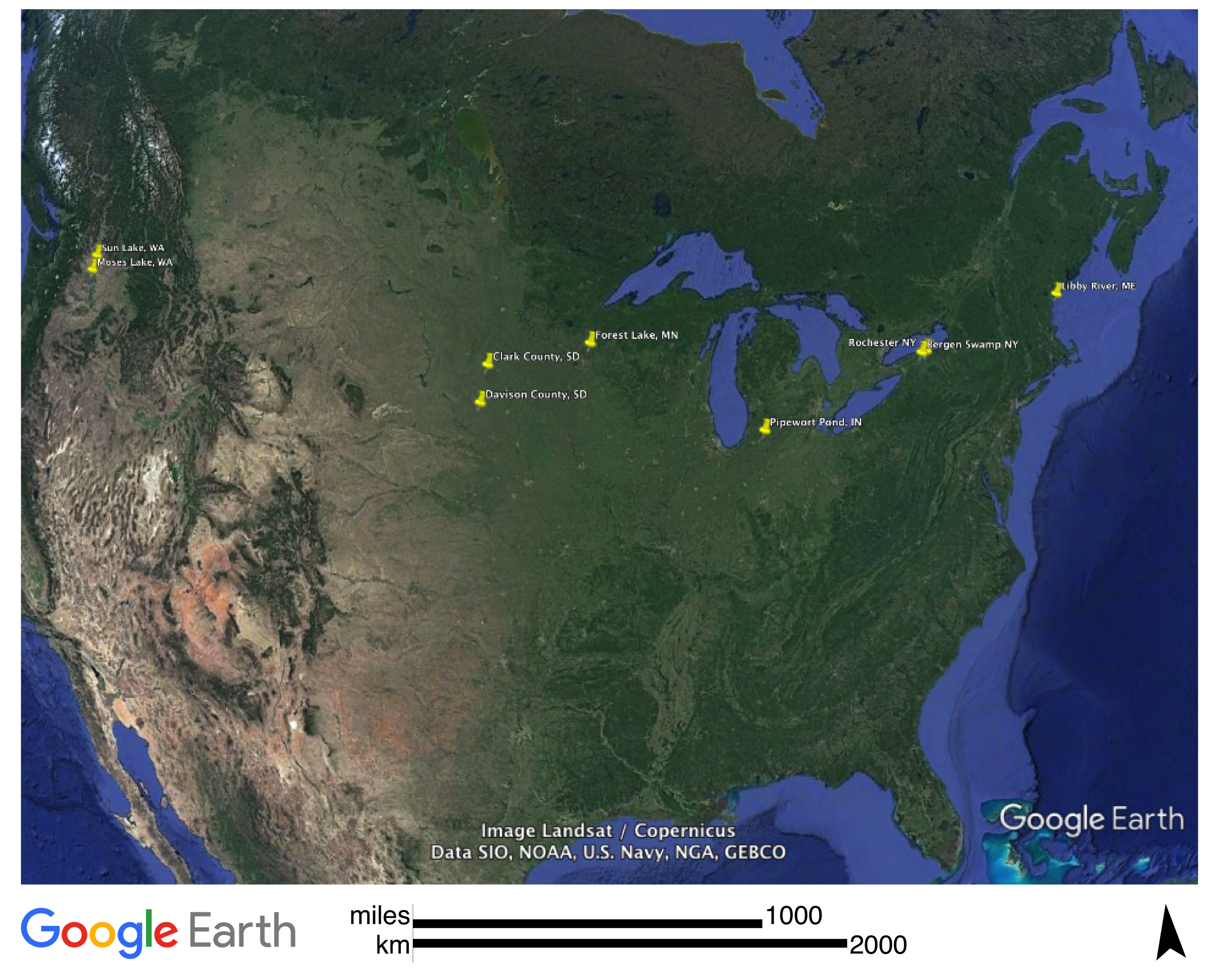
**

**Figure S2.** Early seedling survival (%) of different plant species in control soils and soils conditioned by different populations of either introduced *P. australis* (I) or native *P. australis* *americanus* (N). Two letter code following origin notation indicates US state of original field collection location (see Table S1)

**
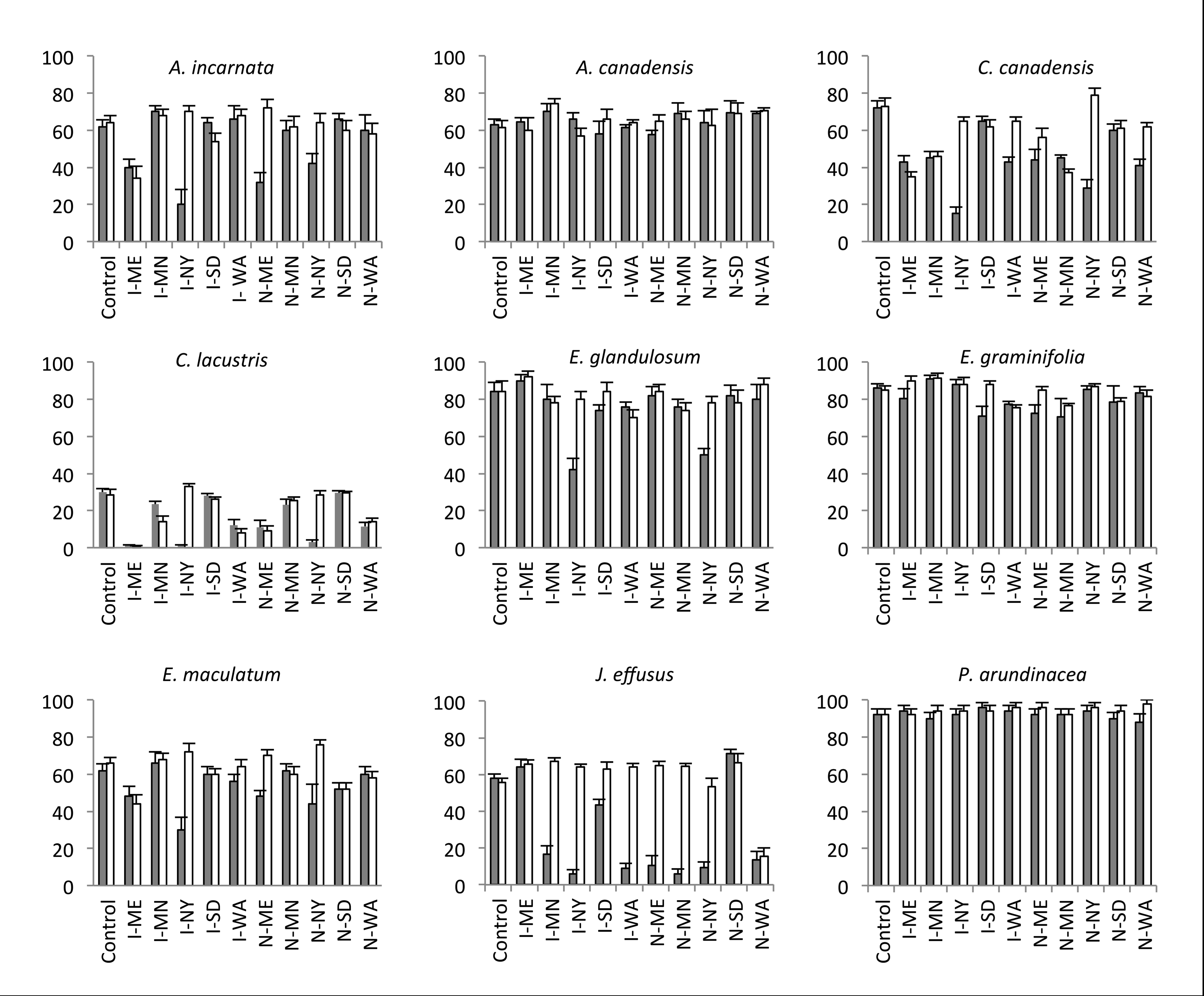
**

Percentage early-seedling survival

**Figure S3.** Probability of transplant survival inside introduced *P. australis* (EU) patches (grey bars) vs in the surrounding mixed wetland plant community (white bars) at four research sites. Data are means ± 1SE of 7 different plant species, each with 20 replicates per site.
